# Negatome 2.0: a database of non-interacting proteins derived by literature mining, manual annotation and protein structure analysis

**DOI:** 10.1093/nar/gkt1079

**Published:** 2013-11-08

**Authors:** Philipp Blohm, Goar Frishman, Pawel Smialowski, Florian Goebels, Benedikt Wachinger, Andreas Ruepp, Dmitrij Frishman

**Affiliations:** ^1^Institute for Bioinformatics and Systems Biology/MIPS, HMGU - German Research Center for Environmental Health, Ingolstaedter Landstrasse 1, 85764 Neuherberg, Germany, ^2^Clueda AG, Elsenheimerstraße 59, 80687 Munich, Germany and ^3^Department of Genome Oriented Bioinformatics, Technische Universitaet Muenchen Wissenschaftszentrum Weihenstephan, 85350 Freising, Germany

## Abstract

Knowledge about non-interacting proteins (NIPs) is important for training the algorithms to predict protein–protein interactions (PPIs) and for assessing the false positive rates of PPI detection efforts. We present the second version of Negatome, a database of proteins and protein domains that are unlikely to engage in physical interactions (available online at http://mips.helmholtz-muenchen.de/proj/ppi/negatome). Negatome is derived by manual curation of literature and by analyzing three-dimensional structures of protein complexes. The main methodological innovation in Negatome 2.0 is the utilization of an advanced text mining procedure to guide the manual annotation process. Potential non-interactions were identified by a modified version of Excerbt, a text mining tool based on semantic sentence analysis. Manual verification shows that nearly a half of the text mining results with the highest confidence values correspond to NIP pairs. Compared to the first version the contents of the database have grown by over 300%.

## INTRODUCTION

Extensive protein interaction maps have been derived for a number of model organisms by modern high-throughput techniques such as yeast two-hybrid assay. While being indispensable tools for systems biology research, these maps are still far from complete and the number of experimentally determined protein–protein interactions (PPIs) continues to grow rapidly, with no saturation in sight. For example, as of this writing the IntAct database (1) lists 48 669 interactions in the human cell, while the total number of human interactions has been estimated to be around 650 000 (2). The overlap between different experimental datasets is also quite poor, indicating that experimental methods possess characteristics biases and capture molecular interactions only partially. This also means that just because two proteins have not yet been reported as interacting does not mean that they actually do not interact in the cell.

Knowledge about non-interacting proteins (NIPs) is as important for training various PPI prediction algorithms as gold standard datasets of positive interactions. It is also indispensable for assessing the false positive rates of PPI detection efforts. However, an experimental method to detect NIPs at proteomic scale remains yet to be invented. A commonly used approach to predict negative interaction data by choosing pairs of proteins that are localized to different cellular compartments has been shown to be biased in terms of the function and amino acid composition of the selected proteins (3). Alternatively, NIPs can also be predicted by randomly selecting any protein pair from a given organism not already known to interact. While straightforward prediction of random pairs may perform poorly in specific biological contexts (3,4), a more intelligent approach has been recently suggested, which only takes into account those protein pairs that were actually tested in a yeast-two-hybrid experiment and not reported to be interacting (5).

In 2009, we made available the first version of a database of mammalian NIP pairs that we call Negatome (6) created by manual curation of literature (1291 negative interactions) and by analyzing protein complexes with known three-dimensional (3D) structure (809 negative interactions). More stringent lists of non-interacting pairs were derived from these two datasets by excluding interactions detected by high-throughput approaches (1162 literature-derived and 745 structure-derived negative interactions, respectively). It spite of the Negatome’s obvious bias toward well-studied cases described in literature and documented by 3D structure analysis (7), it has become a useful tool in PPI analysis and prediction. The Negatome 1.0 dataset has become part of the IntAct database and has also been used to train PPI prediction algorithms (8), classify structural features of interaction interfaces (9), benchmark high-throughput experiments (10,11) and conduct network-based gene function inference (12). By way of an anecdote we are also proud to report that the term Negatome invented by us received the ‘Worst new OMICS word’ award from Jonathan Eisen (http://phylogenomics.blogspot.de/2009/11/worst-new-omics-word-award-negatome.html), although we are not exactly sure why we deserved this honor.

In the 4 years that passed since the publication of Negatome 1.0 the amount of English abstracts in MEDLINE, the primary component of PubMed, has grown by 16.5%, from 10.3 million abstracts in 2009 to 12.0 million abstracts in 2013 (http://www.nlm.nih.gov/bsd/medline_lang_distr.html). The number of 3D protein structures available in the PDB database increased from 62 112 to 93 043 (mid-2013). Here, we present Negatome 2.0, an updated database of high-quality NIP pairs that has been derived by combining text mining and literature curation with protein structure analyses (Table 1). Negatome 2.0 comprises all NIPs from Negatome 1.0 and the additional NIPs that were derived as described in the following.

**Table 1.** Content of the Negatome 2.0 database

Dataset name	Derived from	Description	Number of pairs
PDB	The PDB database	Protein pairs that are members of at least one structural complex but do not interact directly.	4397
PDB-stringent	PDB	The PDB dataset filtered against the IntAct dataset.	4161
PDB-PFAM	PDB-stringent	Non-interacting PFAM domains found in the same structural complex	1234
Manual	Manual literature annotation	Manually annotated literature data describing the lack of protein interaction. High-throughput data are not included.	2171
Manual-stringent	Manual	The Manual dataset filtered against the IntAct dataset.	1991
Manual-PFAM	Manual-stringent	PFAM domain pairs found in the Manual dataset	1453

## DETECTING NEGATIVE PROTEIN INTERACTIONS BY TEXT MINING

The main methodological innovation in Negatome 2.0 is the utilization of the text mining tool Excerbt (13) over the entire corpus of PubMed abstracts and PMC full-text articles (14) to guide the manual annotation effort. In the recent years, advanced text mining systems have been developed that go far beyond mere detection of co-occurring entities. Modern tools such as EVEX (15), BioContext (16) or Excerbt combine syntactic and semantic analyses with rule-based and machine learning approaches. In addition to the extraction of facts about biological events research increasingly focuses on detecting negated facts and speculations (also called hedge detection), as manifested by the inclusion of negation/speculation detection tasks in recent community-wide text mining evaluations (e.g. http://2013.bionlp-st.org) and the emergence of systems for negation detection in molecular events such as Negmole (17) and BioNot (18). A more specialized tool developed by Sanchez-Graillet and Poesio (19) focuses exclusively on negated PPIs.

Excerbt extracts binary relations between biomedical entities using the Senna tool (20). Semantic roles of elements are analyzed, and the so-called predicate-argument-structures (PAS) are derived from each sentence. Excerbt then scrutinizes the agent (who did something) and the theme role (to whom something was done) for entities from an extensive biomedical ontology. For the purposes of Negatome annotation Excerbt was adapted to detect negated PAS (which are normally filtered out). In a first step we selected all PAS that contained proteins in the agent and theme roles and that additionally contained a negation. This process resulted in 58 733 potential non-interactions. Manual verification of a small sample of 20 sentences revealed that 20% of them were actual negative interactions.

To increase the precision of the analysis PAS were restricted to a narrower set of verbs specifically referring to interactions or binding (e.g. ‘to interact’, ‘to bind’, ‘to co-immunoprecipitate’, etc.). This reduced the set of candidate sentences to 2134. In addition, a confidence score was developed based on simple features of the sentence and the PAS:

- Length of the arguments (longer arguments increase the chance of nested structures that were not detected by Senna).
- Length of the sentence (longer sentences are more likely to be wrongly interpreted).
- Type of the relation (some relations such as binding or co-immunoprecipitation have better classification quality than others).
- The word indicating the negation (might be tagged wrongly if not a common negation word is used).
- Whether the agent and the theme are the same entity, which increases likelihood that the text mining system made a mistake. However, in this case the sentence is kept because it is still possible that it contains another non-interaction.

A sample of the results was again evaluated manually. The filter increased the precision of the approach, with more than 50% of the non-interactions classified correctly. The confidence score, in spite of its simplicity, proved to be very informative about the annotation quality. Among the 20 top scoring sentences the precision of text mining was 95%, while for the median 20 and the bottom 20 sentences it was 45% and 15%, respectively.

We found that the main remaining source of errors was the ambiguity of protein symbols and compounds. Since the same names are often used for both kinds of entities, a large number of non-interactions between two compounds as well as between a compound and a protein were detected as protein–protein non-interactions. Attempts to overcome this problem by applying disambiguation algorithms and heuristics at the sentence level proved unsuccessful, probably because proteins and compounds act in a similar fashion and are hence described by similar sentences. We therefore decided to leave the compounds in the dataset and annotate them manually along with the protein–protein non-interactions.

In addition to the precision analysis described above we also examined the recall of our method, i.e. how many of the non-interactions described in the literature were found by Excerbt, by investigating how well Negatome 1.0 can be reproduced by text mining. Only 1 of 40 randomly chosen NIP pairs from Negatome 1.0 was also identified by Excerbt. Around 80% of the cases are currently not reachable for text mining as they are described in the main body of full-text articles not available through PubMed and PMC, in figures and in tables. Five non-interactions from the test sample were not found because Excerbt assigns semantic roles with respect to every verb within a sentence. However, biological events can also be described without using a verb, for example, by using ellipsis, subordinate clauses where the verb is omitted since it can be inferred from the context (e.g. in ‘X interacted with Y, but not with Z’), or by using nominalizations (‘the non-interaction of X and Y’). Furthermore, Excerbt currently does not resolve words, which refer to entities mentioned elsewhere in the text (e.g. pronouns or formulations like ‘these proteins’, known in linguistics as anaphora). In one occasion a term not covered by the Excerbt ontology was used. Only 1 of the 40 sample non-interactions was not found due to an error of Senna. In a further case Excerbt correctly omitted a non-interaction described in the Negatome 1.0, which was found erroneous upon careful inspection.

Since the evaluation described above was dominated by a large amount of non-interactions inaccessible to text mining we analyzed a second test sample of 20 non-interactions from Negatome 1.0 described in the sentences reachable to Excerbt. There were only five non-interactions in this dataset, which could have been potentially found by Excerbt, and three of them were indeed identified while two were missed. One further case was a misclassification of the Negatome 1.0. The remaining 14 cases are not currently addressable by Excerbt as they involve ellipsis (2 cases), anaphora (4 cases), nominalization (2 cases), nested events (1 case), information spread over multiple sentences (1 case) and four protein names not covered by the Excerbt ontology.

Our limited evaluation of the text mining results indicates that there is still considerable potential for increasing the recall (amount of non-interactions found). This can be accomplished by incorporating more sophisticated linguistic analyses capable of resolving more complex sentences. However, the precision (percentage of correctly identified non-interactions in the set of delivered non-interactions) was sufficient to allow for a significant speed up of the manual annotation process.

## MANUAL CURATION AND VERIFICATION OF TEXT MINING RESULTS

The results obtained by text mining were manually verified by examining the original publications, analogously to the annotation of PPIs and protein complexes previously performed by our group (21,22). To facilitate this process, a dedicated annotation tool (available upon request) was implemented. Automatically derived information was supplemented by species names and experimental methods on which the evidence for the absence of an interaction is based. Experimental methods were annotated by the terms from the HUPO-PSI controlled vocabulary for PPIs (23).

**Figure 1.**
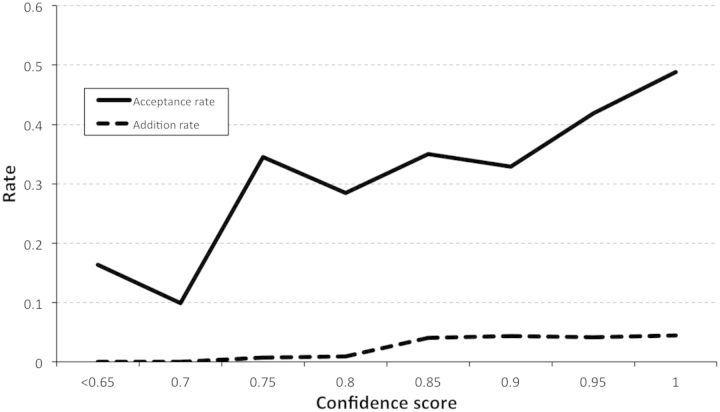
Manual assessment of the text mining performance. The figure shows the number of sentences proposed by the text mining system that were tagged as containing a negative interaction by a human expert (acceptance rate) and the number of negative interactions by the human expert from other sentences stemming from the paper selected by the text mining system (addition rate). Both rates are displayed in relation to the confidence score that was calculated for the text mining results.

Excerbt proposed 2134 protein–protein non-interactions, which were manually annotated. The annotation yielded 895 protein–protein and 119 protein–compound non-interactions. If the annotators came across NIPs while looking for additional information in the articles proposed by Excerbt, e.g. about the used methodology, these NIPs were also annotated. Such additional annotations accounted for ∼10% of the 895 NIPs. As seen in Figure 1 manual verification confirmed the validity of the scores determined by the text mining procedure. Indeed, the acceptance rate of high-scoring hits was significantly higher than that of low scoring sentences. Interestingly, we also found that the scores positively correlated with the rate with which additional non-interactions were added from those sentences not proposed by Excerbt. The dataset obtained by the Excerbt analysis consists to a large extent of mammalian proteins (86%), but information from other organisms was also included. In 64 non-interactions at least one gene product is a splice variant.

## NON-INTERACTING PROTEIN PAIRS AND DOMAINS DERIVED FROM THREE-DIMENSIONAL STRUCTURES

NIP pairs were derived from 3D structures of PDB biological units (24) as described in the original Negatome 1.0 publication (5). Briefly, we selected those PDB chain pairs which were more than 8 Å apart from each other based on Cβ–Cβ(Cα–Cα for glycine) distances. This process yielded 4397 NIP pairs.

## OVERLAP WITH NEGATOME 1.0

Negatome 2.0 contains all 809 structure-based non-interacting pairs from Negatome 1.0, which is expected since we applied the same method to the current PDB database. As for the data generated by manual annotation the overlap between Negatome 1.0 and Negatome 2.0 was limited to only 15 protein pairs due to entirely different approaches taken. In Negatome 1.0, papers containing NIPs were identified based on a simple keyword search in the PubMed database. These papers were then thoroughly studied by biological curators and most of the non-interactions were found in figures and tables. In contrast, in Negatome 2.0, text mining already provided candidate sentences, which were then evaluated by the annotators. Thus, in Negatome 2.0, the focus shifted away from the time-consuming analysis of figures and tables toward a more high-throughput automated approach. The overlap between structure-based and manually annotated NIP pairs is also very low, only 36 pairs. This can be explained by the fact that the structure-based dataset contains *de novo* predictions that have not yet been described in the literature.

## FILTERING AGAINST KNOWN PROTEIN–PROTEIN INTERACTIONS

**Figure 2.**
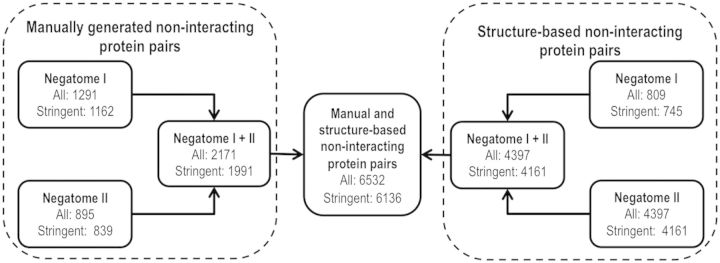
Flowchart explaining how Negatome 2.0 data are generated, merged with Negatome 1.0 and filtered against known interactions to produce stringent datasets.

Analogously to Negatome 1.0 we provide more stringent subsets of our datasets obtained by removing those NIP pairs that are reported as interacting in the IntAct database. As seen in Figure 2, the Manual and Manual-stringent datasets contain 2171 and 1991 pairs, respectively. The structure-based PDB dataset and its stringent version contain 4397 and 4161 pairs, respectively. Combining the manual and the PDB datasets results in a merged dataset of 6532 pairs and a merged stringent dataset containing 6136 pairs. The fraction of non-interacting pairs removed by filtering against IntAct is thus 8%, 5% and 6% for the manual, structure and merged dataset, respectively. In addition, we mapped the Uniprot IDs of the NIP pairs to their PFAM counterparts using the following procedure. First, PFAM domains present in each protein A were identified using the mappings provided by the PDB and SIFTS (25) databases (e.g. A was mapped to A1, A2,…). In the next step, we created all possible domain combinations between the domains constituting two interacting proteins A and B (e.g. A1–B1, A1–B2, A2–B1,…). After that all known interacting domain pairs, as available from 3DID (26), and iPFAM (27), were removed. Using the same procedure we also removed all domain–domain interactions derived from the IntAct database. This filtering step resulted in a PDB-PFAM dataset, which contains 1234 non-interacting domain pairs, and a Manual-PFAM dataset, which contains 1453 pairs.

## FUNDING

DFG International Research Training Group ‘Regulation and Evolution of Cellular Systems’ [GRK 1563]; Funded through the Joint Technology Platform within the Helmholtz Alliance for Systems Biology and the Federal Ministry of Education, Science, Research and Technology [NGFN: 01GR0451, SysMBo, FKZ: 0315494A to B.W.]. Funding for open access charge: Institute for Bioinformatics and Systems Biology/MIPS, HMGU – German Research Center for Environmental Health.

*Conflict of interest statement*. None declared.
